# Structural and Functional Characterization of Recombinant Isoforms of the Lentil Lipid Transfer Protein

**Published:** 2015

**Authors:** I. V. Bogdanov, E. I. Finkina, S. V. Balandin, D. N. Melnikova, E. A. Stukacheva, T. V. Ovchinnikova

**Affiliations:** Shemyakin and Ovchinnikov Institute of Bioorganic Chemistry, Russian Academy of Sciences, Miklukho-Maklaya str., 16/10, 117997 Moscow, Russia

**Keywords:** lipid transfer protein, isoform, lentil, allergen, cross-reactivity, heterologous expression, antimicrobial activity, lipid binding

## Abstract

The recombinant isoforms Lc-LTP1 and Lc-LTP3 of the lentil lipid transfer protein were overexpressed in *E. coli *cells. It was confirmed that both proteins are stabilized by four disulfide bonds and characterized by a high proportion of the α-helical structure. It was found that Lc-LTP1 and Lc-LTP3 possess antimicrobial activity and can bind fatty acids. Both isoforms have the ability to bind specific IgE from sera of patients with food allergies, which recognize similar epitopes of the major peach allergen Pru p 3. Both isoforms were shown to have immunological properties similar to those of other plant allergenic LTPs, but Lc-LTP3 displayed a less pronounced immunoreactivity.

## INTRODUCTION

Plant lipid transfer proteins (LTPs) are a class of small cationic proteins with spatial structures comprising three or four α-helices and stabilized by four disulfide bonds. Hydrophobic cavities in plant LTPs enable them to reversibly bind and transport various lipid molecules [1]. Many proteins of this class possess antimicrobial activity and inhibit the growth of pathogenic bacteria and fungi. LTP synthesis in plants is induced by various stress factors, including attacks by pathogenic microorganisms, draught, excessive soil salinity, etc. [2]. Plant LTPs are believed to be involved in the protection of plants against biotic and abiotic environmental stress factors, in cell wall synthesis, cuticular wax deposition, plant growth modulation, and many other processes [3].

The structure of plant LTPs is highly resistant to thermal denaturation and chemical degradation, as well as to enzymatic cleavage. It is believed that many LTPs, which are highly resistant to degradation by digestive enzymes, are potent allergens responsible for the development of allergic reactions to plant food products [4]. Many LTPs cause latex and pollen allergies. The major peach allergen Pru p 3 is the dominant LTP allergen with a high allergenic capacity. It is involved in the development of allergic cross-reactions to plant foods and pollen [5].

Natural and recombinant allergens, including those belonging to the LTP class, are currently widely used in the development of modern test systems for component- resolved diagnostics. Studies aimed at creating vaccines for preventive allergen-specific immunotherapy (ASIT) on the basis of natural and recombinant allergens are underway [6]. Different isoforms of allergens typically have different immunoreactivities. Therefore, it seems prudent to search for and study isoforms with reduced immunoreactivity, which can be used to develop hypoallergenic variants of major allergens with high clinical effectiveness and low risk of adverse reactions during ASIT [7].

Earlier, we discovered a subfamily of eight lipid transfer proteins (Lc-LTP1-8) in lentil *Lens culinaris* seeds. One of these proteins, namely, Lc-LTP2, was isolated and characterized as a protein possessing antimicrobial activity [8]. It has been demonstrated that Lc-LTP2, like several other plant LTPs, is a food allergen. We have registered it in the IUIS database as Len c 3 [9]. This work focuses on the recombinant production and comparative study of the structural-functional and immunological properties of the two isoforms of lentil LTP: Lc-LTP1 and Lc-LTP3.

## EXPERIMENTAL

**Heterologous expression of LTPs in Escherichia coli cells**

cDNA of lentil *L. culinaris *or peach *Prunus persica *and the following pairs of gene-specific primers were used for PCR amplification of nucleotide sequences encoding the proteins studied: Lc-LTP1 5’-GCGAGATCTATTGATGGAAGAATGGCAATCTCATGCGGAACA- 3’ (forward) 5’-GCGAATTCGCGGATCCTTAGAACCTGATGGTG- 3’ (reverse); Lc-LTP3 5’-GCGAGATCTGATCCGATGGCAGTCTCATGTGGAACT- 3’ (forward) 5’-GCGAATTCGCGGATCCCTTCAAAACTTAATG- 3’ (reverse); Pru p 3 5’-GCGGGATCCATGATAACATGTGGCCAAG-3’ (forward) 5’-GCGGAATTCTCACTTCACGGTGGCGCAGTT-3’ (reverse).

**Fig. 1:**
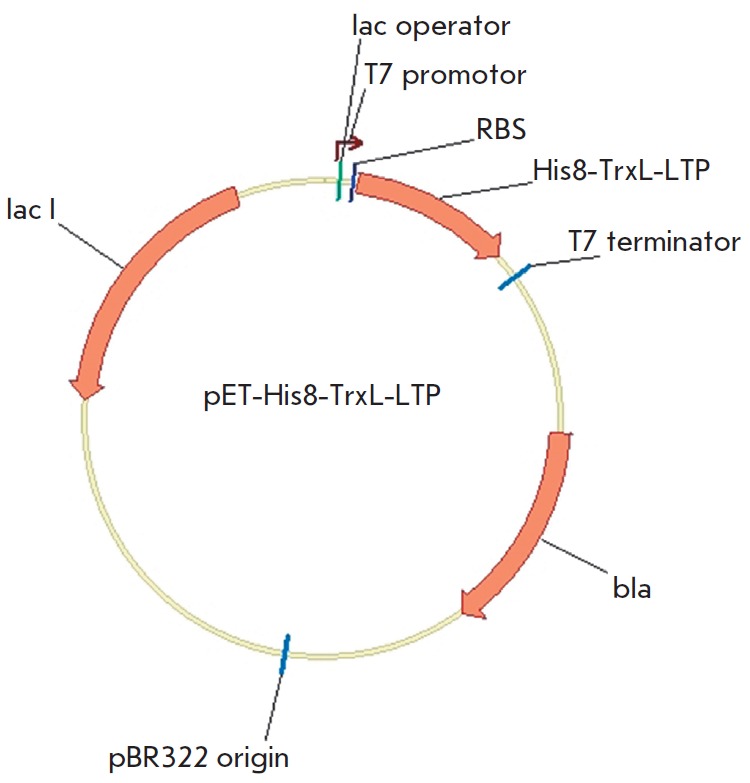
Genetic map of the expression plasmid pET-His8- TrxL-LTP. The plasmid carries a bacteriophage T7 promoter with a *lac *operator, a ribosomal binding site (RBS), the fusion protein coding the sequence His8-TrxL-LTP, where LTP is Lc-LTP1, Lc-LTP3 or Pru p 3, and a T7 terminator;* bla *is the β-lactamase gene; and *lacI *is the lac repressor gene

The expression cassettes carrying a T7 promoter, the ribosomal binding site, a start codon (ATG), sequences encoding histidine octamer and modified thioredoxin A (M37L), cleavage sites (Ile-Asp-Gly-Arg-Met, Asp-Pro-Met or Met) and mature proteins Lc-LTP1 (GenBank AY793553), Lc-LTP3 (GenBank AY793555) and Pru p 3 (GenBank AY792996) were collected after several successive PCR stages and ligated with the BglII/ XhoI fragment of a low-copy-number pET-31b(-) plasmid vector (Novagen) 5.25 kbp in size. Expression plasmids pET-His8-TrxL-Lc-LTP1, pET-His8-TrxL-Lc- LTP3, and pET-His8-TrxL-Pru p 3 (lengths of 6047, 6043, and 6021 bp, respectively) were obtained (*Fig. 1*). These plasmid vectors were used to transform *E. coli *strain BL- 21 (DE3) cells carrying the T7 RNA polymerase gene.

The cells of producing strains were grown in a LB medium containing 50 μg/mL of ampicillin and 20 mM of glucose up to A_600_~0.7. Synthesis of LTP was induced by adding isopropylthio-β-*D*-galactopyranoside to the medium to a final concentration of 0.2 mM. The cells were then grown in 2L flasks with 0.5 L of the nutrient medium for 4–5 h at 25°C (Lc-LTP3 and Pru p 3) or 37°C (Lc-LTP1), using a thermostatic orbital shaker at a stirring rate of 220 rpm.

**Isolation and purification of recombinant LTPs**

Cell pellets obtained by centrifugation were resuspended in buffer A (50 mM Tris-HCl, 0.5 M NaCl, 20 mM imidazole, pH 7.8) containing 1 mM phenylmethylsulfonyl fluoride at a ratio of 1:5 (v/v). The cells were destroyed on ice in an ultrasonic homogenizer using eight cycles of 45 s. The clarified cell lysate obtained by centrifugation was used for Lc-LTP3 and Pru p 3 purification. Metal chelate chromatography was performed on a Ni2+sepharose column at a flow rate of 0.7 mL/min. The fusion proteins were eluted with buffer A containing 0.5 M imidazole. Lc-LTP1 was isolated from inclusion bodies, which were washed twice with buffer A containing 1% Triton-X100 and then solubilized in buffer A containing 6 M guanidine hydrochloride. Metal chelate chromatography was performed in the same buffer system containing 6 M guanidine hydrochloride. All eluates were dialyzed against 3 L of acidified water (pH 3.0) at 4°C overnight. The resulting dialysates were freeze-dried. The fusion proteins were cleaved with cyanogen bromide. For this, the fusion proteins were first dissolved in 80% TFA in a concentration of 10–20 mg/mL. A 100-fold molar excess of cyanogen bromide was then added, and the mixture was incubated in the dark at room temperature for 16–20 h. The reaction was stopped by adding a threefold volume of water. The samples were then evaporated in a vacuum concentrator. The target proteins were purified by repeated metal chelate chromatography on the same column in the same buffer system containing 6 M guanidine hydrochloride. The final purification of the recombinant proteins was performed on a Reprosil-Pur C18-AQ column (Dr. Maisch GmbH) in the presence of 0.1% TFA at a flow rate of 2 mL/min in a gradient of acetonitrile concentration from 5 to 80% over 60 min. Refolding of the purified Lc-LTP1 was performed in a buffer (50 mM Tris-HCl, pH 8.0, 20 mM NaCl, 0.8 mM KCl, 1 mM EDTA) containing 1 M urea, 0.8 M L-arginine, and 2 mM GSH/0.2 mM GSSG [10]. For the refolding, the recombinant protein was dissolved in this buffer to a concentration of 0.1 mg/mL, incubated at 4°C overnight and purified by RP-HPLC on a Luna C18 column (Phenomenex) in the presence of 0.1% TFA using a gradient of acetonitrile concentration from 5 to 80% over 60 min. The fractions obtained at different isolation stages were analyzed by SDS-PAGE (15% gel) in the Tris-glycine system according to Laemmli [11].

**Mass spectrometry, Edman microsequencing and CD spectroscopy**

The molecular weights of the recombinant LTPs were determined using a Reflect III MALDI-TOF mass spectrometer (Bruker) equipped with a UV laser (336 nm). The amino acid sequence was determined using the Procise cLC 491 protein sequencing system (Applied Biosystems). Circular dichroism spectra were recorded at room temperature using a J-810 spectropolarimeter (Jasco) in a cell with an optical path of 0.01 cm in a wavelength range of 180–250 nm (scan rate 1 nm) using aqueous solutions of the recombinant proteins at a concentration of 1 mg/mL.

**Sera and antibodies**

Sera from allergic patients (n = 20) were collected at the Center of Molecular Diagnostics at the Central Research Institute of Epidemiology. Out of them, we selected the sera from nine patients allergic to plant products, which contained IgE specific to recombinant Pru p 3. Sera samples from nonallergic individuals were used as a negative control. Total IgE levels in the sera of allergic patients were determined using a Total IgE HRP EIA kit (Dr. Fooke) according to the manufacturer’s instructions.

Polyclonal anti-Lc-LTP2-antibodies were prepared by immunization of rabbits. At the first stage of preparing a hyperimmune serum, the rabbits were subcutaneously administered recombinant Lc-LTP2 (150 μg/ rabbit) with complete Freund’s adjuvant, then a half dose of the antigen with incomplete Freund’s adjuvant, and finally the recombinant protein in PBS. Polyclonal anti-Lc-LTP2-antibodies were purified by fractional precipitation of proteins with ammonium sulfate. Sera samples obtained from the same rabbits prior to immunization were used as a negative control.

**Immunoblotting**

A lentil seed extract was prepared as described in [8]. Following SDS-PAGE (15% gel), the proteins were electrotransferred to a nitrocellulose membrane in a buffer containing 20% of methanol and 0.1% of SDS. At the first stage, the membrane was incubated in a 1% solution of nonfat dry milk in TBS. The membrane was washed with TBST and incubated in a solution of polyclonal rabbit anti-Lc-LTP2-antibodies in a 1% milk solution in TBS (dilution 1:200) for 2 h at room temperature. After washing, the membrane was incubated in a solution of goat anti-rabbit IgG-horseradish peroxidase conjugated antibodies (Sigma) in a 1% milk solution in TBS for 1 h at room temperature. The membrane washed with TBST was then treated with a TMB solution for membranes (Sigma). The enzymatic reaction was terminated by washing the membrane with water to remove residual substrate.

**Enzyme-linked immunosorbent assay (ELISA)**

The recombinant LTPs (0.5 μg) were added to the wells of a 96-well plate (Costar) in 50 μL of TBS and incubated for 1 h at 37°C. After washing with the same TBST buffer solution, the plate was incubated at 37°C for 2 h with a 1% solution of BSA in TBS. The plate was then incubated at 37°C for 2 h with sera from allergic patients prepared by serial dilutions (1:2–1:16) in TBS. After washing with TBST, a solution of goat anti-human IgE-horseradish peroxidase conjugated antibodies (Sigma) was added to the wells and the plate was incubated at 37°C for 1 h. Bound antibodies were detected after washing the wells with TBST using TMB for ELISA (Sigma). The enzymatic reaction was stopped by adding 4N H_2_SO_4_. The resulting data were analyzed by measuring the absorbance in the wells at 450 nm.

For ELISA with polyclonal rabbit anti-Lc-LTP2 antibodies, free binding sites were blocked under the same conditions and the plate was incubated with a solution of polyclonal rabbit anti-Lc-LTP2 antibodies in TBS (1:500—1:64,000 dilutions) at 37°C for 1 h. After washing with TBST, a solution of goat anti-rabbit IgG-horseradish peroxidase conjugated antibodies in TBS was added to the wells and the plate was incubated at 37°C for 1 h. Detection was also performed using TMB.

In ELISA inhibition assays, smaller amounts of the recombinant proteins (0.2 μg) were used for coating and the patients’ sera were pre-incubated with serial dilutions of the recombinant Pru p 3 at a concentration of 0.02-200 μg/mL at 37°C for 3 h.

**Antimicrobial activity**

The bacteria *Agrobacterium tumefaciens *A281,* Clavibacter michiganensis *Ac-1144, and *Pseudomonas syringae *B-1546 were inoculated into a liquid LB medium and incubated at 30°C under constant stirring until A_600_ = 1.0–1.5. The test fungi *Aspergillus niger *F-2259,* Fusarium solani *F-142, *Alternaria alternata *F-3047,* Botrytis cinerea *F-3700, and *Neurospora crassa *F-184 were grown on potato sucrose agar at room temperature until active sporulation. Aliquots (110 μL) of the bacterial cultures (4×10^4^ CFU/mL) or spore suspension (104 spores/mL) in the culture medium and 10 μL of sterile protein solutions of different concentrations in 0.1% TFA were added to the wells of a 96-well plate. Each version of the test was performed in triplicate. The plate was incubated in a thermostatic shaker at 30°C. Culture growth was assessed by measuring the absorbance in the wells at 620 nm. 0.1% TFA was used as a negative control. The protein concentrations ensuring 50% inhibition of culture growth (IC_50_) were determined after 24 or 48 h bacterial or fungal culture incubation, respectively. Spore germination and hyphal morphology were evaluated using a CKX41 light inverted microscope (Olympus) after 12 and 24 h spore incubation in a liquid culture medium with the protein solutions.

**Fatty acid binding**

Fluorescence spectra were recorded using an F-4000 spectrofluorimeter (Hitachi) at 25°C. The spectral width of the slit of the monochromator excitation and emission was 5 nm. TNS fluorescence was excited at 320 nm and recorded in the 330–450 nm range. The maximum fluorescence intensity was detected at 437 nm. A TNS solution in a concentration of 3 μM in a buffer solution (175 mM *D*-mannitol, 0.5 mM K_2_SO_4_, 0.5 mM CaCl_2_, 5 mM MES, pH 7.0) with or without stearic acid (to a concentration of 65 μM) was incubated in a cuvette for 1 min under constant stirring. Fluorescence spectra were subsequently recorded. Recombinant LTPs were then added to a concentration of 2.5 μM, incubated for 2 min, and fluorescence spectra were recorded [12]. The results are expressed as a percentage of the fluorescence intensity of the protein-TNS complex according to the formula ((F – F_0_)/F_C_) × 100%, where F_0_ is the fluorescence intensity of TNS in the solution; F and F_C_ are the fluorescence intensities of the protein-TNS complex either with or without the lipid added, respectively.

## RESULTS AND DISCUSSION

Lipid transfer proteins in plant genomes are represented by gene families encoding different LTP isoforms. Multiple isoforms of the lipid transfer protein have been detected in a single plant, thus giving grounds for more in-depth research of the biological role of each of them. It was suggested that the expression of certain LTP isoforms is primarily regulated by the environment and that the synthesis of multiple LTP isoforms is an element of the plant defense system against a variety of abiotic and biotic stresses [13]. This assumption was confirmed in studies of the differential gene expression of LTP isoforms in various organs and tissues of plants under abiotic and biotic stress conditions using sesame [14], arabidopsis [15], pepper [16], the castor oil plant [17], grapes [18], and tomato [19]. It was shown that biosynthesis of certain LTP isoforms in plants is tissue-specific and that the genes of certain isoforms are expressed at different stages of plant ontogeny.

 Recently we found eight LTPs in germinated lentil seeds, named Lc-LTP1–8 [8]. It was shown that biosynthesis of the isolated isoforms of the lentil lipid transfer protein Lc-LTP2,4,7,8 occurs during early development of seedlings and may take place due to the involvement of these proteins in plants protection against pathogens or in lipid transport during the active metabolism phase during germination. The biological role of the isoforms Lc-LTP1,3,5,6 remains unclear.

The structural and functional properties of one of the lentil LTPs, namely, Lc-LTP2, were studied in detail. The spatial structure of this protein is typical of that of a representative of the LTP class comprising four α-helixes [20]. Hydrophobic amino acid residues in the protein are pointed inwards and form a hydrophobic cavity capable of hosting lipid ligands. Lc-LTP2 binds lipids, exhibits antimicrobial activity, and is a major lentil allergen registered as Len c 3 [9].

**Fig. 2:**
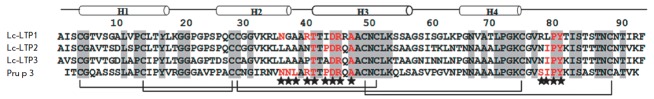
Comparison of the amino acid sequences of lentil LTP isoforms and the major peach allergen Pru p 3. Conservative amino acid residues are shown in gray. The disulfide bonds are shown as brackets. α-Helical regions of Lc-LTP2 are shown above [20]. The amino acid residues included in the conformational epitopes of Pru p 3 are shown in red with stars [21]

This work focuses on the isolation and comparative study of the structural-functional and immunological properties of two other lentil LTP isoforms. The isoforms Lc-LTP1 and Lc-LTP3 chosen for a comparative study differ most significantly from Lc-LTP2 in terms of their amino acid sequence (72 and 77% homology, respectively) (*Fig. 2*). These proteins consist of 93 amino acid residues, including eight conservatively located cysteine residues. Their isoelectric points lie in the alkaline pH range (9.53 and 8.32 for Lc-LTP1 and Lc-LTP3, respectively). These proteins contain amino acid residues comprising conformational epitopes of the dominant LTP class allergen, peach Pru p 3, which suggests that the two lentil LTP isoforms possess allergenic properties.

**Isolation and characterization of recombinant lentil LTP isoforms**

The recombinants Lc-LTP1 and Lc-LTP3 were prepared in a manner similar to that described for Lc- LTP2 [9]. Isolation and purification of the recombinant proteins were performed using soluble (Lc-LTP3) and insoluble (Lc-LTP1) cellular fractions and involved several steps. During the expression, the fusion protein His8-TrxL-Lc-LTP3 was predominantly accumulated in its soluble form in the cytoplasm. His8-TrxL-Lc- LTP1, which has an isoelectric point lying at a higher pH, was accumulated in both soluble and insoluble forms. However, the solution primarily contained the His8-TrxL-Lc-LTP1 forms with a truncated C-terminal region, which may have been formed by proteolytic cleavage of Lc-LTP1. Therefore, expression of the fusion protein was carried out at a higher temperature and it was isolated from the insoluble cell fraction. The fusion proteins were purified by metal chelate chromatography in either nondenaturing or denaturing conditions using step gradient elution with imidazole. The fusion proteins were cleaved with cyanogen bromide in an acidic medium, and the reaction products were separated by repeated metal chelate chromatography. The final purification of the recombinant proteins was performed by RP-HPLC.

**Fig. 3:**
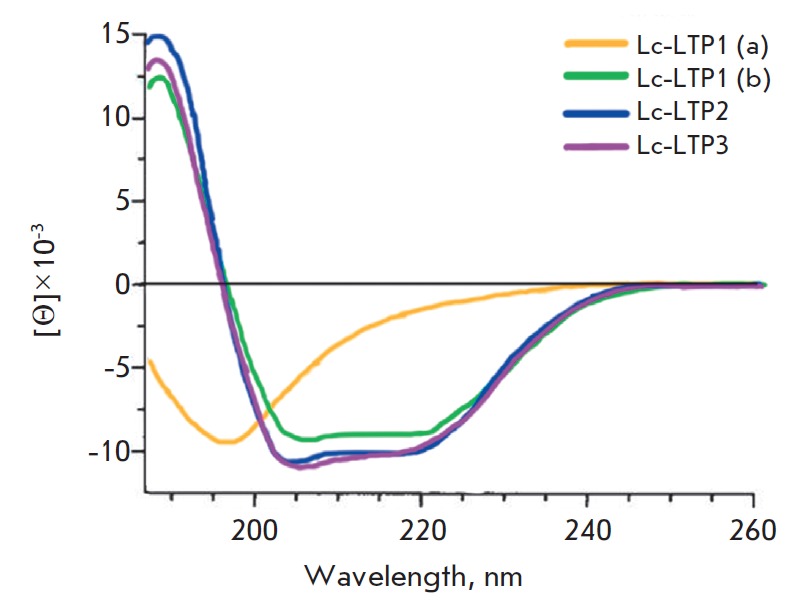
CD spectra of lentil LTPs. (a), (b) – CD spectra of the recombinant Lc-LTP1 before and after refolding, respectively

The secondary structure of the recombinant proteins was studied by CD spectroscopy. The CD spectrum of Lc-LTP3 was similar to that of Lc-LTP2 and featured a curve typical of proteins with a high content of *α*-helical structures. The CD spectrum of recombinant Lc-LTP1 had a different shape and suggested that the protein is not structured (*Fig. 3*). Therefore, purified Lc-LTP1 was refolded at low temperature under mild denaturing conditions in the presence of 1 M urea, L-arginine, which prevents protein aggregation, and a pair of oxidized and reduced glutathiones. The reaction products were separated by RP-HPLC. An analysis of the Lc-LTP1 CD spectrum after refolding showed that the protein assumed the conformation typical of plant LTPs.

**Fig. 4:**
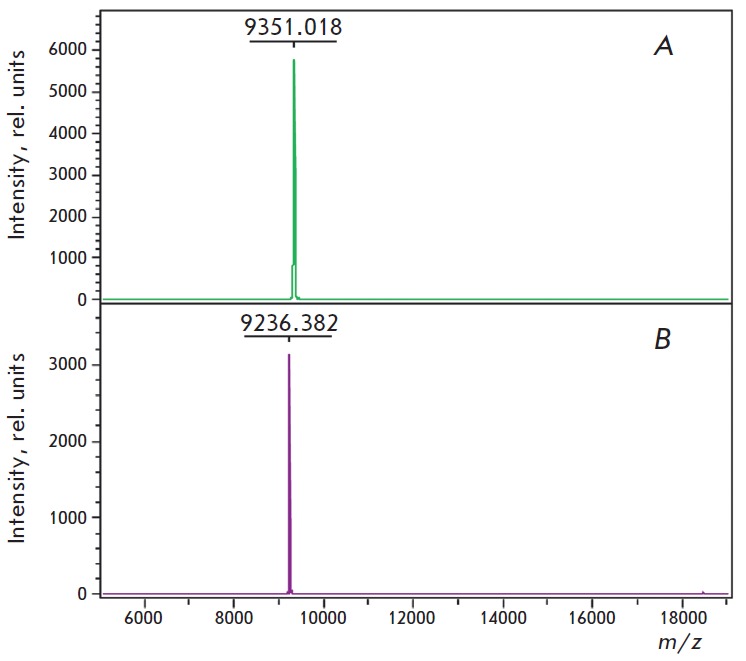
MALDI-TOF mass spectra of the recombinant Lc- LTP1 (a) and Lc-LTP3 (b)

The recombinant protein samples were analyzed by SDS-PAGE. It was shown that in the absence of β-mer- captoethanol, Lc-LTP1 and Lc-LTP3 exist both in dimeric and monomeric forms, which is typical of LTPs [9]. Addition of a reducing agent leads to cleavage of disulfide bonds and dimer dissolution. Homogeneity and the identity of the recombinant LTPs and natural proteins were confirmed by MALDI-TOF mass spectrometry and automated Edman microsequencing. A mass spectrometric analysis showed that the molecular weights of the recombinant Lc-LTP1 (*m/z *of 9351.02) and Lc-LTP3 (*m/z *of 9236.38) correspond to the calculated weights of LTPs whose structures are stabilized by four disulfide bonds (9350.93 and 9235.65 Da, respectively) (*Fig. 4*). The measured *m/z *values correspond to the masses of protonated molecular ions [M+H]+. The yields of the recombinant proteins were no lower than 3 and 5 mg/L of the culture, based on pure Lc-LTP1 and Lc-LTP3, respectively. Recombinant Pru p 3 was prepared in the same manner as Lc-LTP3, and its yield was 4 mg/L of the culture.

**Functional activity of the recombinant lentil LTP isoforms**

It is well known that many members of LTPs possess antimicrobial activity and the cytoplasmic membrane is the intended target of their antimicrobial action [22]. It is believed that cationic plant LTPs interact with the anionic components of the cytoplasmic membrane, which leads to its destabilization and disruption of permeability [20].

**Table 1:** Antimicrobial activity of the recombinant lentil LTPs

Microorganism	IC_50_, μM		
	Lc-LTP1	Lc-LTP2	Lc-LTP3
Bacteria			
Agrobacterium tumefaciens	40	20–40	40
Clavibacter michiganensis	40	>40	>40
Pseudomonas syringae	>40	>40	>40
Fungi			
Alternaria alternata	40	>40	40
Aspergillus niger	5–10	10	10
Botrytis cinerea	20–40	10–20	>40
Fusarium solani	20–40	>40	40
Neurospora crassa	40	20–40	20–40

The comparative study of the antimicrobial activity of the three lentil LTP isoforms was conducted using the Gram-negative bacteria *A. tumefaciens *and *P. syringae* and Gram-positive bacterium *C. michiganensis*, as well as the fungi *A. alternata*, *A. niger*, *B. cinerea*, *F. solani*, and *N. crassa*. It was demonstrated that the recombinant Lc-LTP1 and Lc-LTP3, as well as Lc-LTP2, also exhibit antifungal and mild antibacterial activity and their antimicrobial action is nonspecific (*Table 1*). *A. niger *culture, the black rot plant pathogen, was the one most susceptible to all three proteins. It was shown that recombinant Lc-LTP1 and Lc-LTP3, as well as Lc- LTP2, inhibit spore germination of phytopathogenic fungi, mycelium growth and development, but do not affect the hyphal morphology. No significant difference in the strength of antimicrobial activity was observed for the three lentil LTP isoforms, despite the marked differences in the primary structures and acid-base properties of these proteins. Based on this, it was suggested that it is not solely the nonspecific electrostatic interaction between LTP and the membrane that is responsible for the antimicrobial effect.

In addition to antimicrobial activity, virtually all known plant LTPs have the ability to bind and transport a variety of lipids. The LTP structure has a hydrophobic cavity capable of binding hydrophobic molecules. Plant LTPs bind a wide range of ligands, including fatty acids with a C10–C18 chain length, acyl derivatives of coenzyme A, phospho- and galactolipids, prostaglandin B2, molecules of organic solvents, and certain drugs [23]. The effectiveness of the binding of various lipid ligands depends on the size of the hydrophobic cavity in the protein. It is believed that LTPs participate in many processes in plants via their ability to bind and carry various lipids.

**Fig. 5:**
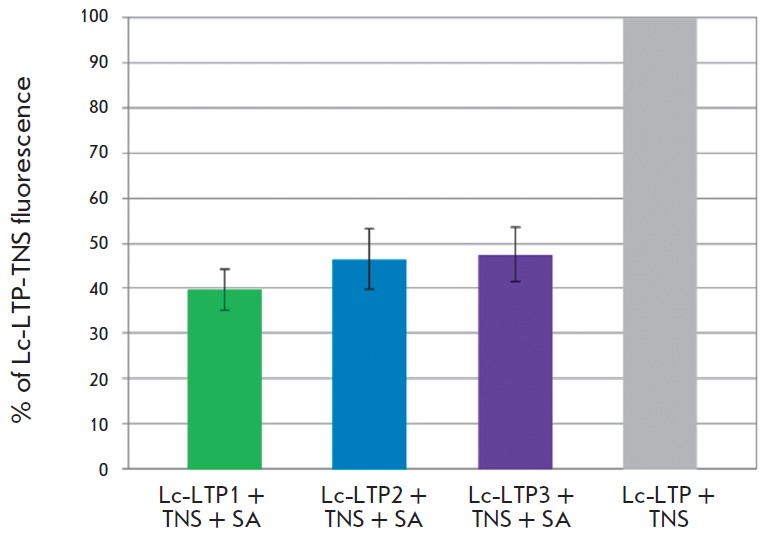
Effect of stearic acid (SA) on the fluorescence level of the LTP-TNS complexes

NMR spectroscopy has previously been used to show that the recombinant Lc-LTP2 has a hydrophobic cavity allowing it to interact with dimyristoylphosphatidylglycerol [20]. In this work, we studied the interaction of the three recombinant lentil LTPs with stearic acid using a fluorescent TNS probe, whose fluorescence increases in a hydrophobic environment. It was shown that addition of misfolded Lc-LTP1 (without prior refolding) to a TNS solution does not affect the intensity of its fluorescence. The fluorescence intensity of TNS, however, significantly increases after the addition of recombinant Lc-LTP2 and Lc-LTP3, as well as Lc- LTP1 after the refolding. This implies that protein-TNS complexes are formed, and a hydrophobic cavity capable of binding hydrophobic molecules is present in the structures of all three LTPs. The addition of each of the three recombinant lentil LTPs to a TNS–stearic acid mixture resulted in a less significant increase in the fluorescence intensity and was indicative of the competition between fatty acid molecules and TNS for binding sites in the proteins (*Fig. 5*). Thus, it was demonstrated that all three isoforms possess the ability to bind fatty acids. No significant difference in the effectiveness of fatty acid binding by the three proteins was observed, which can be attributed to the similar sizes of the hydrophobic cavities in the structures of the three lentil LTPs.

**Immunological properties of the recombinant lentil LTP isoforms**

LTPs from various plants have been characterized as allergens. Quite often, allergic reactions are caused by cross-reactivity between the major LTP allergen, peach Pru p 3, and homologous allergenic proteins from different plant foods and pollens. We have previously shown that lentil Lc-LTP2, registered in the IUIS database as Len c 3, is a food allergen. This allergen binds to specific IgE in sera of patients with food allergies, which recognize epitopes similar to those of the major peach allergen Pru p 3 so that it may cause allergic cross-reactions [9]. In this work, we have conducted a comparative study of the immunological properties and cross-reactivity of the two other lentil LTP isoforms. Polyclonal rabbit anti-Lc-LTP2 antibodies and sera of patients with food allergies containing specific IgE to the recombinant Pru p 3 were used in the study.

**Fig. 6:**
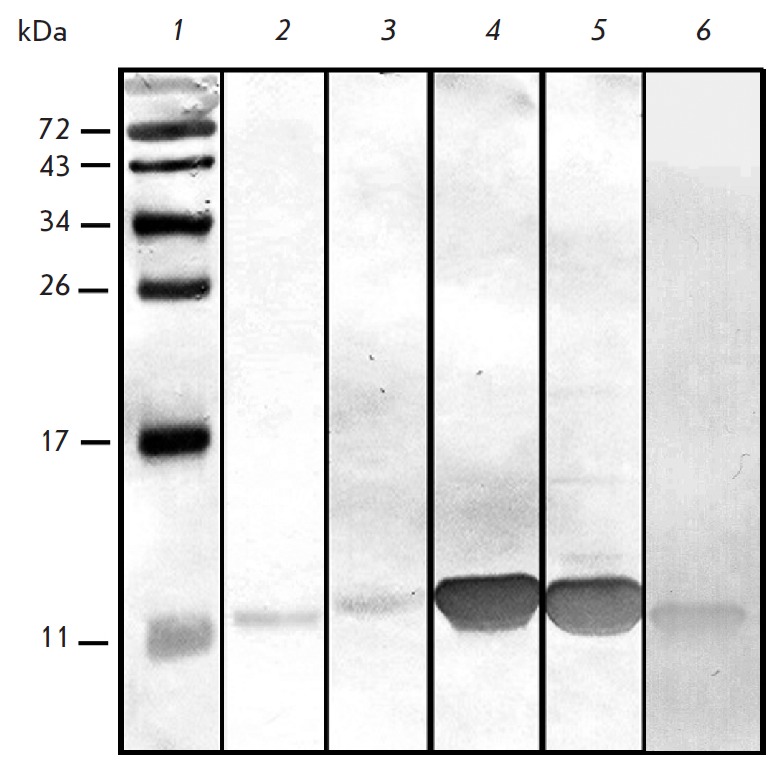
Immunoblotting with rabbit polyclonal anti-Lc- LTP2 antibodies: 1 – molecular mass standards; 2 – the lentil seed extract; 3–5 – the recombinant Lc-LTP1,2,3, respectively; 6 – the recombinant Pru p 3

**Fig. 7:**
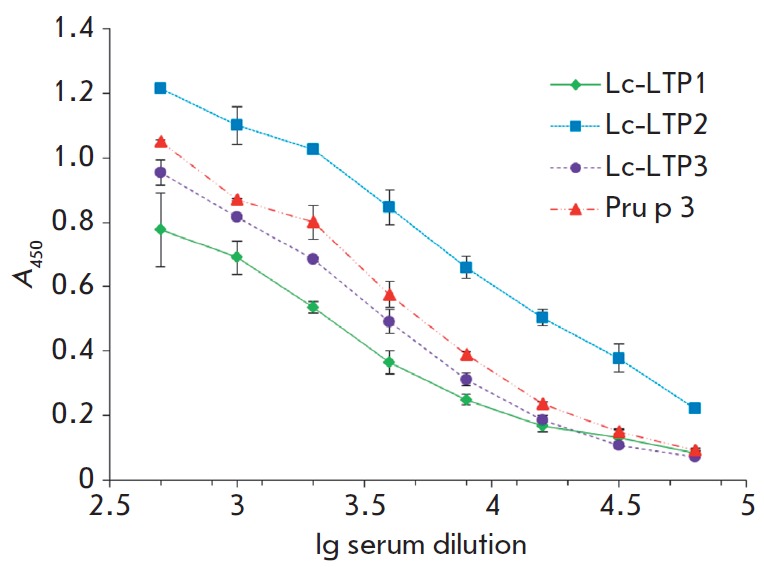
ELISA with rabbit polyclonal anti-Lc-LTP2 antibodies

The interaction of rabbit polyclonal anti-Lc-LTP2 IgG with three lentil LTP isoforms and peach Pru p 3 was investigated by immunoblotting (*Fig. 6*) and ELISA (*Fig. 7*). The results of immunoblotting with pre-reduced recombinant LTPs demonstrated that polyclonal rabbit anti-Lc-LTP2 antibodies bind to all three lentil LTP isoforms and peach Pru p 3. As one would expect, the highest level of binding of anti-Lc-LTP2 antibodies was observed in the case of Lc-LTP2. The lowest one was observed in the case of Lc-LTP1, although, peach Pru p 3 is the least structurally similar to Lc-LTP2 (55% homology). The ELISA results were fundamentally the same as the results of immunoblotting, even though we used native proteins in this test. The maximum efficiency of binding to anti-Lc-LTP2 antibodies was observed for Lc-LTP2; while the lowest, for Lc-LTP1. The results of immunoblotting and ELISA reveal a similar structural organization of all LTPs and at least a partial similarity of their linear and conformational antigenic determinants.

The ability of recombinant proteins to bind to specific IgE in sera of patients allergic to fruits, nuts, and beans was demonstrated by ELISA (*Table 2*). All three lentil LTP isoforms bind to specific IgE, but the analysis of the majority of sera from the patients showed that their immunoreactivity was lower than that of Pru p 3. IgE-immunoreactivity of the recombinant Lc- LTP3 was lower than that of the two other isoforms. This indicates that all three isoforms of lentil LTPs have allergenic properties and according to the preliminary data, Lc-LTP3 is the least allergenic lentil LTP isoform. The less pronounced immunoreactivity of Lc-LTP3 may be attributed to the fact that it contains fewer amino acid residues constituting conformational epitopes of peach Pru p 3 (*Fig. 2*) compared to other lentil LTPs (7 of 13).

**Table 2:** Characterization of sera from patients with food allergies

No.	Sex (M/F)	Total IgE, IU/mL	Specific IgE* (ELISA), A_450_				Allergenic products
			Lc-LTP1	Lc-LTP2	Lc-LTP3	Pru p 3	
1	M	794	1.4 ± 0.09	1.26 ± 0.19	1.28 ± 0.1	1.35 ± 0.14	Nuts
2	F	556	0.7 ± 0.12	0.77 ± 0.11	0.84 ± 0.07	0.85 ± 0.05	Nuts
3	M	525	1.08± 0.04	1.08 ± 0.11	1.07 ± 0.02	1.09 ± 0.03	Nuts
4	F	479	0.53± 0.05	0.57 ± 0.03	0.37 ±.03	0.82 ± 0.05	Sesame, soy beans
5	M	417	0.28± 0.01	0.4 ± 0.02	0.32 ± 0.06	0.27 ± 0.01	Nuts, fruits
6	F	407	0.77± 0.07	0.93 ± 0.02	0.29 ± 0.08	1.01 ± 0.07	Nuts, tomatoes
7	F	302	0.33± 0.03	0.37 ±0.05	0.31 ± 0.1	0.33 ± 0.02	Nuts
8	M	71	0.6 ± 0.02	0.62 ± 0.02	0.4 ± 0.01	0.81 ± 0.02	Pea
9	F	25	0.46± 0.05	0.53 ± 0.14	0.45 ± 0.04	0.64 ± 0.02	Nuts

*Note. Data were obtained using 1:2 serum dilutions.

**Fig. 8:**
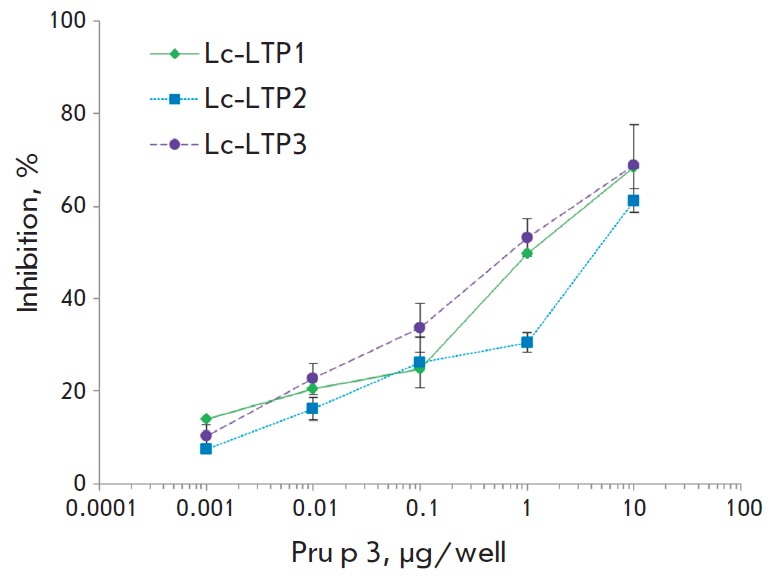
Effect of the recombinant Pru p 3 on lentil LTP binding to specific IgE from the serum of patient no.3 with food allergy

Cross-reactivity of the recombinant lentil LTP isoforms was investigated by ELISA using the recombinant Pru p 3 as an inhibitor of IgE-binding (*Fig. 8*). Inhibition of IgE-binding was observed for all three lentil LTPs. The results indicate that the isoforms Lc-LTP1, Lc-LTP3, and Lc-LTP2 contain epitopes that are similar to the major peach allergen Pru p 3.

## CONCLUSIONS

In this study, we have obtained the recombinant isoforms of the lentil lipid transfer proteins Lc-LTP1 and Lc-LTP3 and conducted a comparative study of the structural-functional and immunological properties of the four lipid transfer proteins. Despite significant differences in the amino acid sequences of the three isoforms of lentil LTPs, their functional properties were quite similar. It was shown that all three proteins contain α-helical regions and are characterized by the presence of a hydrophobic cavity that ensures their ability to bind fatty acids. All isoforms of lentil LTPs possess antimicrobial activity characterized by low specificity. The search for natural ligands of different lentil LTP isoforms, as well as identification of the factors affecting the induction of their biosynthesis, will pave the way for a deeper understanding of the functional role of the multiplicity of LTP isoforms.

At the same time, the study revealed certain differences in the immunoreactivity of the three lentil LTP isoforms. It was shown that Lc-LTP1 and Lc-LTP3, as well as Len c 3 deposited in the IUIS allergen database, are able of binding specific IgE from sera of patients with food allergies, recognizing epitopes similar to those of the major peach allergen Pru p 3. However, the immunoreactivity of Lc-LTP3 was less pronounced than that of the other isoforms. Further research into the structural organization and allergenic properties of Lc-LTP3 will reveal the key amino acid residues whose replacement leads to a decreased immunoreactivity of plant LTPs. It will also create conditions for the development of hypoallergenic variants of lipid transfer proteins and their use for allergen vaccination.

## Abbreviations

ASIT: allergen-specific immunotherapy
TNS: 2-(p-toluidino)-6-naphthalenesulfonic acid
BSA: bovine serum albumin
ELISA: enzyme-linked immunosorbent assay
GSH and GSSG: reduced and oxidized glutathione
LTP: lipid transfer protein
MES: 2-(N-morpholino)ethanesulfonic acid
PBS: phosphate-buffered saline
PBST: phosphate-buffered saline containing Tween-20
TBS: Tris-buffered saline
TBST: Tris-buffered saline containing Tween-20
TMB: 3,3’,5,5’-tetramethylbenzidine
